# Involvement of Auxin and Brassinosteroid in Dwarfism of Autotetraploid Apple (*Malus* × *domestica*)

**DOI:** 10.1038/srep26719

**Published:** 2016-05-24

**Authors:** Yue Ma, Hao Xue, Lei Zhang, Feng Zhang, Chunqing Ou, Feng Wang, Zhihong Zhang

**Affiliations:** 1 College of Horticulture, Shenyang Agricultural University, 120 Dongling Road, Shenyang 110866, China; 2 Research Institution of Pomology, Chinese Academy of Agricultural Sciences, Xingcheng, Liaoning 125100, China; 3 College of Plant Protection, Shenyang Agricultural University, 120 Dongling Road, Shenyang 110866, China

## Abstract

The plant height is an important trait in fruit tree. However, the molecular mechanism on dwarfism is still poorly understood. We found that colchicine-induced autotetraploid apple plants (*Malus* × *domestica*) exhibited a dwarf phenotype. The vertical length of cortical parenchyma cells was shorter in autotetraploids than in diploids, by observing paraffin sections. Hormone levels of indoleacetic acid (IAA) and brassinosteroid (BR) were significantly decreased in 3- and 5-year-old autotetraploid plants. Digital gene expression (DGE) analysis showed that the differentially expressed genes were mainly involved in IAA and BR pathways. microRNA390 was significantly upregulated according to microarray analysis. Exogenous application of IAA and BR promoted stem elongation of both apple plants grown in medium. The results show that dwarfing in autotetraploid apple plants is most likely regulated by IAA and BR. The dwarf phenotype of autotetraploid apple plants could be due to accumulation of miR390 after genome doubling, leading to upregulation of apple *trans-acting short-interfering RNA 3* (*MdTAS3*) expression, which in turn downregulates the expression of *MdARF3*. Overall, this leads to partial interruption of the IAA and BR signal transduction pathway. Our study provides important insights into the molecular mechanisms underlying dwarfism in autopolyploid apple plants.

Polyploids are organisms with three or more complete sets of chromosomes per nucleus. All of the angiosperms have ancient polyploids with experiencing one or more rounds of genome duplication during their evolution^1^. Polyploid plants can be classified according to the origin of their doubled genomes: In autopolyploids, the same parental set is multiplied, whereas in allopolyploids, doubling involves different parental sets^2^,^3^,^4^,^5^. Studies on the growth and biochemical characteristics of natural polyploid plants have shown that polyploids have several advantages over their diploid counterparts, such as larger vegetative organs, a higher metabolic rate, more secondary metabolites, and increased stress resistance^2^,^6^. Thus, the use of polyploids is becoming increasingly common in plant breeding^2^,^7^,^8^,^9^. *In vitro* induction of autotetraploid plants has become an effective tool in plant breeding programmes^10^. Autotetraploid lines of many plant species exhibit the typical morphological characteristics of polyploids; for example, tetraploid *Nicotiana attenuata, N. obtusifolia*^11^ and *Lolium*^12^ plants have enlarged corolla limbs, larger seeds and longer leaves compared with the corresponding diploids.

Changes in the characteristics of polyploids are mainly caused by differences in gene expression. In allopolyploids, gene expression changes can be attributed to the nature of divergent genomes and possible interactions between various genetic components^9^,^13^,^14^. However, changes in gene expression in autopolyploids are not the same as those in allopolyploids and can be attributed to chromosome dosage or epigenetic changes in the duplicated genome^15^. Some studies have suggested that mild changes in gene expression may be related to phenotypic alterations^5^,^16^,^17^. At present, there are few reports describing the systematic analysis of a group of genes in an autopolyploid.

In our preliminary study, autotetraploid plants were induced *in vitro* from the apple (*Malus* × *domestica*) cultivar ‘Hanfu’ using colchicine-treated leaf explants. We found that the autotetraploid plants exhibited dwarf characteristics during five years of observation after transplantation in the field. This result is not consistent with observations of other herbaceous polyploids, which are typically larger than diploids. The plant height has relationship with cell elongation and cell division which are regulated by plant hormones. Gibberellic acid (GA), auxin and brassinosteroid (BR) are three important hormones well known to control plant growth and development^18^,^19^,^20^. The expression change of key genes in these hormone regulation pathways could also lead plant dwarfism.

Here, we used digital gene expression (DGE) and quantitative reverse transcription-polymerase chain reaction (qRT-PCR) techniques to compare gene expression profiles between diploid and autotetraploid apple plants. Microarray and qRT-PCR techniques were used to analyse expression levels of microRNAs (miRNAs). An HPLC-ESI-MS/MS system was employed for plant hormone analysis. The results revealed that dwarfism in the autotetraploid apple plants may be regulated by auxin and BR. This study provides important and unexpected insights into autotetraploid dwarfism.

## Results

### Dwarfism of autotetraploid apple

**Figure 1:**
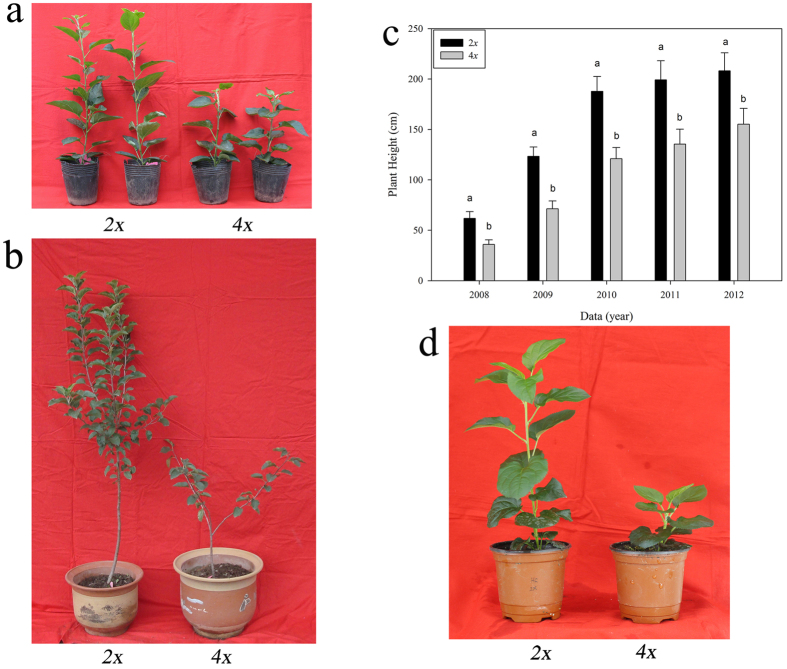
Transplanted autotetraploid and diploid plants of the apple cultivar ‘Hanfu’. Transplants in 2008 (**a**) and 2009 (**b**), plant height at five years (**c**), transplants derived from repeated shoot culture (**d**).

Diploid and autotetraploid apple plants (cultivar ‘Hanfu’) were cultured *in vitro* and subsequently transferred to pots in a solar greenhouse in March of 2008. Both the diploid and autotetraploid plants flowered and produced fruit from 2010 (3 years old) to 2012 (5 years old). After 5 years of cultivation in the field, all of the autotetraploid plants were morphologically distinguishable from the diploids. Specifically, the autotetraploids exhibited a dwarf phenotype (Fig. 1a–c). We next investigated plant height in autumn after growth cessation of new shoots from 2008 to 2012. As shown in Table 1, in 2002, the average plant height of the diploids was 208.20 ± 2.92 cm, while the average height of the autotetraploids was 155.20 ± 6.69 cm; thus, the autotetraploid plants were significantly shorter than the diploid plants. The average growth increases of the diploid and autotetraploid plants were 61.60 cm and 35.50 cm, respectively, during 2009 and 64.48 cm and 49.60 cm, respectively, during 2010. Thus, the autotetraploid plants were shorter than the diploid plants due to the decreased growth rate after transplantation. To exclude the possibility that colchicine poisoning caused the dwarf phenotype of autotetraploid apple plants, we cultured the shoot tips from these 5-year-old diploid and autotetraploid apple plants *in vitro*, and we transplanted the plants into pots at the same time. After 5 months of observation, we found that the autotetraploid apple plants were still significantly shorter than the diploids (Fig. 1d). Thus, after repeated verification, we confirmed that the dwarf phenotype of autotetraploid apple plants is a real phenomenon and could not be caused by colchicine poisoning.

### Histological observation of shoot segments

**Figure 2:**
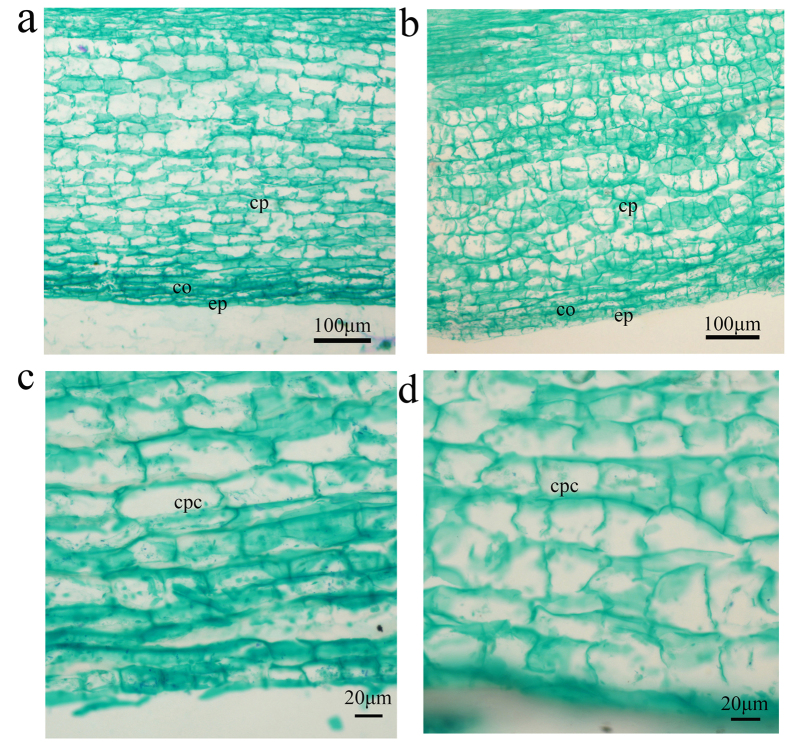
Longitudinal sections of young stem cortices from autotetraploid and diploid apple plants. (**a**,**c**) diploid apple; (**b**,**d**) autotetraploid apple. ep, epidermis; cp, cortical parenchyma; cpc, cortical parenchyma cell.

To further explore the cause of autotetraploid dwarfism, we dissected the shoot of an autotetraploid plant and a diploid plant. We found that the length of the autotetraploid cells was significantly reduced (Fig. 2a,b). The average cell lengths in the autotetraploid and diploid were 37.35 ± 0.74 μm and 59.35 ± 3.13 μm, respectively (Fig. 2c). The autotetraploid cell length was 1.6 times shorter than that of the diploid. The average cell widths in the autotetraploid and diploid were 40.81 ± 0.49 μm and 29.08 ± 1.00 μm, respectively (Fig. 2d). The autotetraploid cell width was 1.3 times larger than that of the diploid.

### DGE analysis of apple libraries

**Figure 3:**
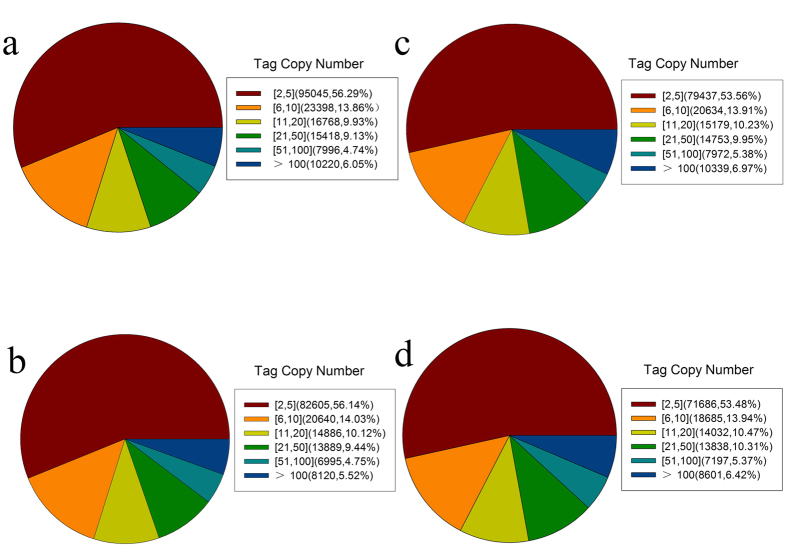
Distribution of distinct clean sequence tags. Diploid samples 2*x*–1 (**a**) and 2*x*–2 (**b**) and autotetraploid samples 4*x*–1 (**c**) and 4*x*–2 (**d**) are shown.

To examine gene expression in the autotetraploid dwarf apple plants, we performed DGE. We used the Solexa Genome Analyzer to perform high-throughput tag-sequencing (Tag-seq) analysis of poly(A)-enriched RNAs from libraries constructed from young shoots of both autotetraploid and diploid apple (cv. ‘Hanfu’) plants. Two biological replicates were performed for both autotetraploid and diploid plants, and >4.7 million tags were obtained from each. The number of tags with distinct sequences ranged from 3.1 to 3.5 million (Table 2). The distribution of total and distinct tag counts over different tag abundance categories was similar among these four libraries. Among the distinct tags, >5% had a copy number >100, >30% had copy numbers between 6 and 50, and >50% of the transcripts had copy numbers between 2 and 5 (Fig. 3). After removal of low-quality tags, we obtained a total of 5,788,249 (2*x*–1), 4,539,994 (2*x*–2), 5,757,002 (4*x*–1) and 4,761,962 (4*x*–2) clean tags that corresponded to 168,845 (2*x*–1), 147135 (2*x*–2), 148,314 (4*x*–1) and 134,039 (4*x*–2) distinct tags, respectively (Table 2). In this study, all clean tags were aligned to the NCBI database and the reference apple genome database (http://www.rosaceae.org/).

To identify genes involved in plant dwarfism, we used EBseq software^21^ to compare the transcripts in diploid and autotetraploid samples. We normalized the read density measurement and used a false discovery rate (FDR) < 0.001 and | log_2_ Ratio | ≥1 as thresholds to determine the statistical significance of gene expression changes. A total of 3,158 genes were differently expressed between the diploid and autotetraploid libraries. Of these, 1,036 were upregulated and 2,122 were downregulated in the autotetraploid library (Online Resource 1).

**Figure 4:**
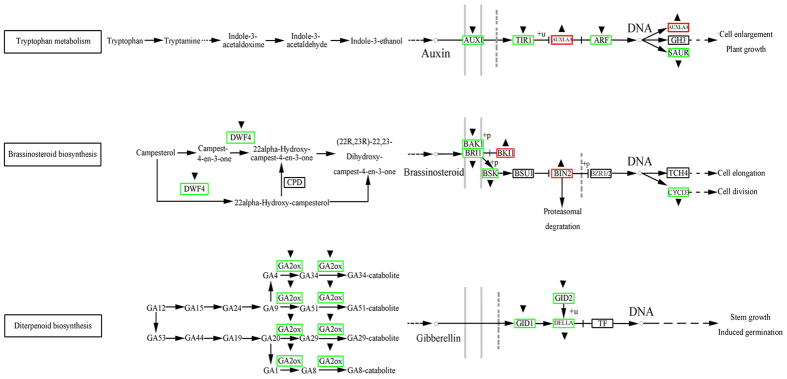
Differential expression of genes involved in hormone biosynthesis and signal transduction pathways in diploid and autotetraploid apple plants. Genes marked with “▴” and “▾” were either upregulated or downregulated, respectively, in the autotetraploids.

To understand the functions of differentially expressed genes, we mapped these genes to terms in the Kyoto Encyclopedia of Genes and Genomes (KEGG) database. The differentially expressed genes were found to be involved in 113 pathways. Notably, we observed specific enrichment of genes in pathways involved in plant hormone signal transduction (map 04075). In searching for pathways with a potential relationship to dwarfism, we devoted specific attention to genes from the auxin, GA, and BR biosynthesis and signal transduction pathways. We found that most of the differentially expressed genes from these pathways were significantly downregulated in the autotetraploid library (Table 3, Fig. 4). Examples of genes that were downregulated include *AUXIN-RESISTANT1* (*AUX1*, MDP0000885425) (log_2_^(4*x*/2*x*) ^= −1.81), *auxin response factor 3* (*MdARF3*, MDP0000179650) (log_2_^(4*x*/2*x*)^ = −1.17), *DWF4* (MDP144510) (log_2_^(4*x*/2*x*)^ = −1.17), *brassinosteroid signalling kinase* (*BSK*, MDP0000092692) (log_2_^(4*x*/2*x*) ^= −1.96), *gibberellin 2-oxidase gene* (*GA2ox*, MDP0000137705) (log_2_^(4*x*/2*x*)^ = −6.92), *DELLA* (MDP0000205622) (log_2_^(4*x*/2*x*) ^= −1.54), and *GIBBERELLIN-INSENSITIVE DWARF2* (*GID2*, MDP0000126528) (log_2_^(4*x*/2*x*) ^= −1.62), while expression of *BRl1 kinase inhibitor 1* (*BKI1*, MDP0000124873) (log_2_^(4*x*/2*x*)^ = 2.03) and *brassinosteroid insensitive 2* (*BIN2*, MDP0000295137) (log_2_^(4*x*/2*x*) ^= 1.61) was upregulated (Table 3).

### Verification of DGE analysis results by qRT-PCR

**Figure 5:**
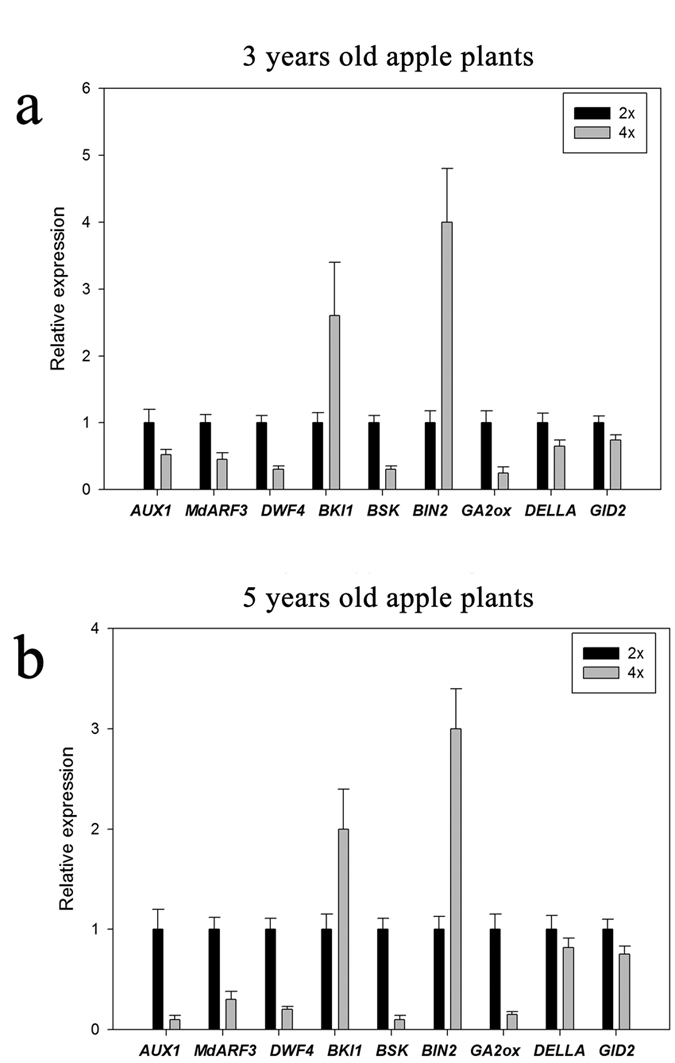
Differentially expressed genes between diploid and autotetraploid apple plants as determined by qRT-PCR. The relative expression levels of each gene are shown for the diploid (2*x*) and autotetraploid (4*x*) plants. (**a**) Gene expression differed between 2*x* and 4*x* 5-year-old apple plants. Using the 2*x* plants as a control, 0.52-fold downregulation was observed for *AUX1*, 0.45-fold for *MdARF3*, 0.3-fold for *DWF4*, 0.35-fold for *BSK*, 0.25-fold for *GA2ox*, 0.65-fold for *DELLA* and 0.74-fold for *GID2* in the 4*x* plants; however, 2.6-fold upregulation of *BKI1* and 4-fold upregulation of *BIN2* were observed in 4*x* plants. (**b**) Gene expression differed between 2*x* and 4*x* 5-year-old apple plants. Using the 2*x* plants as a control, 0.1-fold downregulation was observed for *AUX1*, 0.3-fold for *MdARF3*, 0.3-fold for *DWF4*, 0.2-fold for *BSK*, 0.15-fold for *GA2ox*, 0.82-fold for *DELLA* and 0.75-fold for *GID2* in the 4*x* plants; however, 2-fold upregulation of *BKI1* and 3-fold upregulation of *BIN2* were observed in the 4*x* plants.

To confirm the results of DGE analysis, the expression levels of nine genes involved in plant hormone biosynthesis and signal transduction pathways (*AUX1, ARF3, DWF4, BKI1, BSK, BIN2, GA2ox, DELLA* and *GID2*) were measured by qRT-PCR in 3-year-old and 5-year-old diploid and autotetraploid apple plants (Fig. 5). The results obtained for all of these genes agreed with the results obtained with DGE analysis. However, *DELLA* and *GID2* did not show significant differences in gene expression between autotetraploids and diploids.

### Microarray analysis of miRNAs

**Figure 6:**
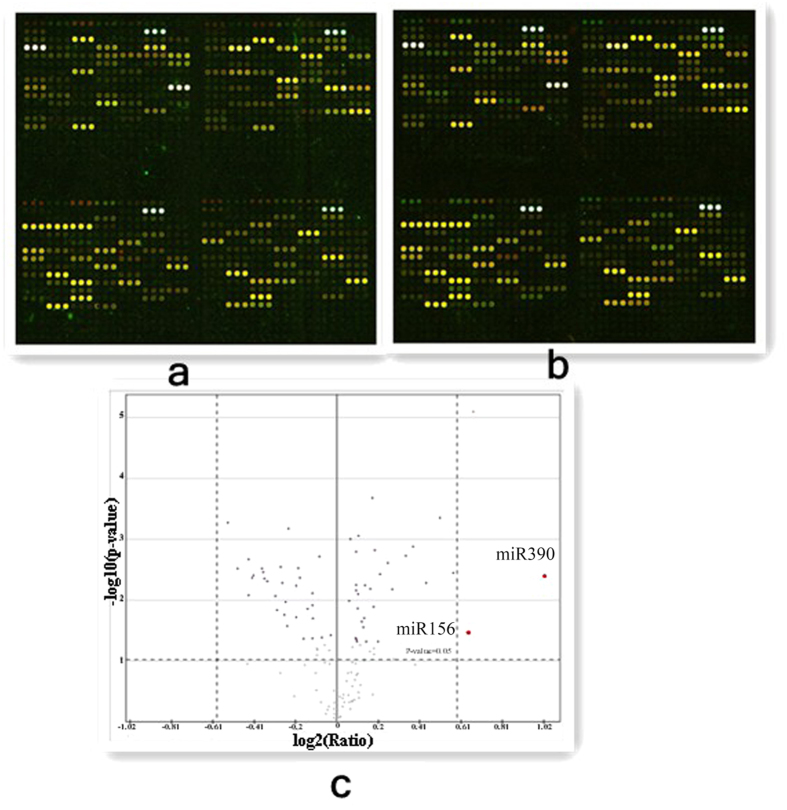
Double-fluorescence scanning image of the miRNA microarray. (**a**) Autotetraploid (4*x*) apple labeled with Cy5 and diploid (2*x*) apple labeled with Cy3. (**b**) Autotetraploid (4*x*) apple labeled with Cy3 and diploid (2*x*) apple labeled with Cy5.

Changes in the gene expression of autopolyploids are not the same as those in allopolyploids and can be attributed to chromosome dosage or epigenetic changes in the duplicated genome^15^. Small RNAs are commonly involved in the epigenetic regulation of gene expression. Plant miRNAs are small, endogenous, noncoding RNAs generated from the processing of local hairpin precursor structures. Mature miRNAs can target mRNAs for cleavage, leading to the destabilization of target mRNAs and thereby suppressing specific gene expression^22^,^23^. We used miRNA microarrays to assess the differential expression of miRNAs in diploids and autotetraploids. The microarrays were labeled with Cy3 and Cy5 (Fig. 6a,b). A total of 154 miRNAs were checked because the intensity values of their fluorescent signals were above 400 in both of the microarrays. Differential expression analysis of miRNAs was performed using SAM software. Only miR390, which regulates the formation of trans-acting siRNA (TAS3 ta-siRNA), was significantly upregulated, with a log_2_^(4*x*/2*x*)^ ratio of 1.02 (Fig. 6c).

### Assessment of differences in the expression of miR390 and *MdTAS3* in apple by qRT-PCR

**Figure 7:**
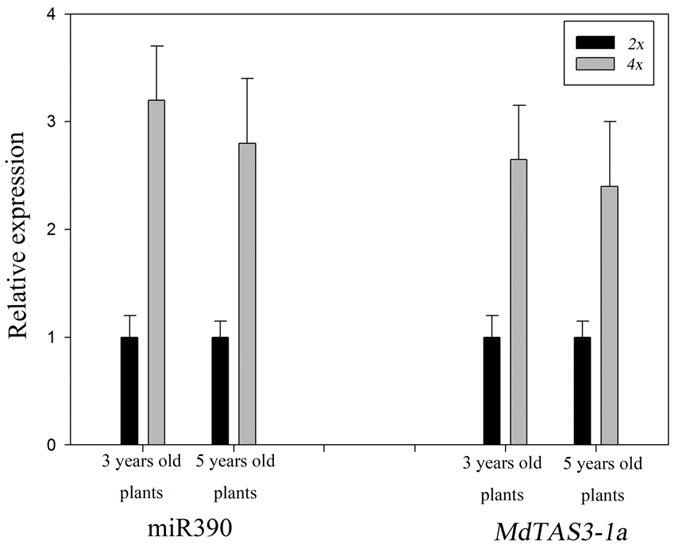
Expression of miR390 and MdTAS3-1a in diploid and autotetraploid 3- and 5-year-old apple plants as determined by qRT-PCR. The relative expression levels of each gene are shown for the diploid (2*x*) and autotetraploid (4*x*) plants. Using the 2*x* plant as a control, miR390 expression was found to be upregulated by 3.2-fold in 3-year-old 4*x* apple plants and upregulated by 2.65-fold in 5-year-old 4*x* apple plants. Using the 2*x* plant as a control, MdTAS3-1 expression was found to be upregulated by 2.4-fold in 3-year-old 4*x* apple plants and upregulated by 2.8-fold in 5-year-old 4*x* apple plants.

In apple, miR390 is an indirect negative regulator of *MdARF3* via *MdTAS3* ta-siRNAs^24^. Interestingly, miR390 was upregulated in the microarray analysis of autotetraploid apple. We used qRT-PCR to analyse the differences in miR390 and *TAS3-1a* expression between diploids and autotetraploids using 3-year-old and 5-year-old plants. Both miR390 and *TAS3-1a* were found to be significantly upregulated in the autotetraploids (Fig. 7). In apple, miR390 is an indirect negative regulator of *MdARF3* via *MdTAS3 ta-siRNAs*^24^. The expression of *MdARF3* was significantly downregulated in autotetraploid apple based on both DGE and qRT-PCR analysis (Table 3, Fig. 5).

### Differences in endogenous hormone concentrations between diploid and autotetraploid apple plants

To determine the relationship between plant height and endogenous hormone concentrations, we also compared the relative levels of Indoleacetic acid (IAA), GA_1_, and BR in the shoot tips of diploid and autotetraploid 3-year-old and 5-year-old plants. The results are shown in Table 4. The levels of IAA and BR in the autotetraploids were significantly lower than those in diploid plants of both ages.

### Effects of exogenous supplementation of tissue culture plants with IAA and epicastasterone

**Figure 8:**
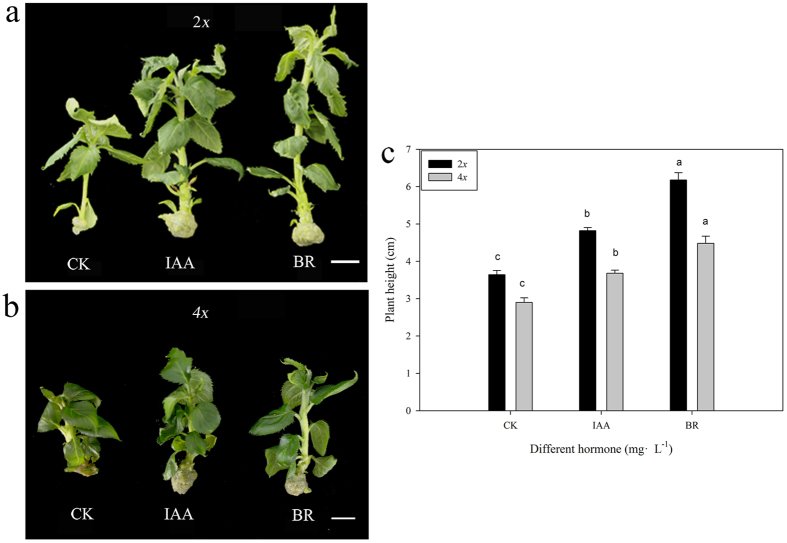
Tissue culture plants exposed to exogenous hormones. (**a,b**) Morphological differences in diploid and tetraploid apple plants grown in tissue culture with exogenous hormone supplementation. (**c**) Differences in the heights of diploid and tetraploid apple plants grown in tissue culture with exogenous hormone supplementation.

IAA and BR are two important hormones that are well known to control plant height^18^,^19^,^20^. To determine the relationship between plant height and exogenous supplementation with IAA and epicastasterone (EBR), we added these two hormones to shoot proliferation medium and performed shoot culture. Each treatment included 5 bottles, and each bottle contained 2 plants (Fig. 8a,b). Five typical plants were selected from each treatment for plant height measurement. Changes in plant height under each hormone treatment were measured. The plant height of autotetraploids was increased by 54.48% under BR treatment and by 26.90% under IAA treatment compared with the control. The plant height of diploids was increased by 69.98% under BR treatment and by 32.42% under IAA treatment compared with the control (Fig. 8c). Exogenous supplementation with IAA and EBR significantly increased the height of both autotetraploid and diploid apple plants (Fig. 8a,c). EBR had stronger effect on plant height (Fig. 8c,e).

## Discussion

### Dwarfism of autotetraploid apple is not caused by colchicine poisoning

Over a period of five years (2008–2012), comparative analysis of the heights of diploid and autotetraploid apple plants revealed that the autotetraploids were significantly shorter than the diploids (Fig. 1a,c; Table 1). This result was similar to results reported in studies of autotetraploid *Dendranthema nankingense* and *Paulownia tomentosa* plants^25^,^26^. However, it is contrary to the early belief that polyploidization often results in larger organs^27^,^28^. Colchicine poisoning is one possible cause of the dwarf phenotype of autotetraploid plants after genome doubling^29^. To exclude the possibility that colchicine poisoning caused the dwarf phenotype of autotetraploid apple, we cultured shoot tips from 5-year-old diploid and autotetraploid apple plants *in vitro*. Then, both diploid and autotetraploid plants grown in tissue culture were transplanted into pots at the same time. After 5 months of observation, the autotetraploid apple plants were still significantly shorter than the diploids (Fig. 1d). Thus, after repeated verification, we confirmed that the dwarf phenotype of autotetraploid apple plants is a real phenomenon and could not be caused by colchicine poisoning. Evidence obtained from examinations of paraffin sections suggested that the autotetraploid dwarfism phenomenon was related to significant reductions in the length of cells in the plant stem (Fig. 2). Phenotypic differences are caused by changes in gene expression. The available evidence concerning the mechanisms underlying gene expression changes in polyploids suggests that these gene expression changes are due to epigenetic variation^7^,^8^,^9^,^15^,^30^. To understand the molecular mechanism by which hormones affect plant growth, we attempted to determine the relationship between differentially expressed genes and the autotetraploid dwarf phenotype. When examining the DGE data, we devoted more attention to the key genes involved in the biosynthesis and signal transduction pathways of these three plant hormones (Fig. 4).

### The relationship between GA and the autotetraploid dwarf phenotype

*GA20ox, GA3ox*, and *GA2ox* are three key genes encoding enzymes that catalyse the later reactions in the GA biosynthesis pathway^31^. Plants with loss-of-function mutations in *GA20ox* and *GA3ox* display a dwarf phenotype. On the other hand, overexpression of the *GA2ox* gene also produces dwarf plants^32^. *GA2ox* decreases the levels of active GAs in plants^33^. In our DGE analysis, we found that *GA20ox* and *GA3ox* showed no significant expression differences between diploids and autotetraploids (Fig. 4). GA_1_ is known as the main growth-active GA, while the other GAs are not active until they are converted to GA_1_^19^,^34^,^35^. Studies of maize have shown that tall plants have a higher GA_1_ level, while GA_1_ is absent or present at a very low level in dwarf plants^36^. In sorghum, tall genotypes also showed a 2–6-fold increase in GA_1_ concentration compared with short genotypes^18^. It should also be noted that GA_1_ is mainly present in the youngest internodes of the stem^35^. The content of endogenous GA_1_ may be positively related to internode length in peas^34^. In this study, we did not find significant differences in GA_1_ content between autotetraploid and diploid plants (Table 4). This result supports our finding that there were no significant differences in the expression of genes involved in the GA biosynthesis pathway.

*DELLA* is a key gene in the GA signal transduction pathway, and *DELLA* deletion mutations in maize and *Brassica napus* exhibit a dwarf phenotype^37^,^38^. The expression of *DELLA* and *GID1* was not significantly different between diploids and autotetraploids based on qRT-PCR (Fig. 5a,b).

Taken together, our data show that genes involved in GA biosynthesis and the GA signal transduction pathway were not differentially expressed between autotetraploids and diploids. Therefore, GAs are not likely to be involved in the dwarf phenotype of autotetraploids.

### The relationship between BR and the autotetraploid dwarf phenotype

*DWF4* is a key gene in the BR biosynthesis pathway^39^. Overexpression of *DWF4* in *Arabidopsis dwf4* mutants has been shown to rescue the dwarf phenotype^40^. Therefore, in our study, the significant downregulation of *DWF4* in autotetraploid apple plants (Fig. 5a,b) could lead to a decrease in the BR level and a dwarf phenotype.

Previous research has indicated that application of EBR promotes plant elongation; in fact, five BR dwarf mutants in *Arabidopsis* were previously shown to be rescued by BR feeding (*det2*^41^, *cpd*^42^, *dwf1*^43^, *ste1*^44^, and *sax1*^45^). The *dumpy* mutant of tomato, which exhibits reduced BR content and a dwarf phenotype, can be rescued by application of BRs^46^. Transgenic overexpression of the *UGT73C5* gene was shown to decrease BR content and cause dwarfism; however, a wild type phenotype was restored by exogenous treatment with 24-epibrassinolide^47^. In this study, we observed a significant decrease in BR content in autotetraploids (Table 4). Exogenous application of BR significantly promoted plant elongation (Fig. 8). Thus, the decreased BR content in autotetraploids could cause the dwarf phenotype observed in these plants.

*BKI1* and BIN2 are two key genes involved in the BR signal transduction pathway^39^. *BKI1* encodes a BRI1 kinase inhibitor, which is a negative regulator of brassinosteroid signalling. Overexpression of *BKI1* in *Arabidopsis* resulted in dwarf plants. These plants had smaller rosettes, a reduced stature, reduced petiole length, more rounded rosette leaves, and delayed flowering compared with wild type^48^. Therefore, in our study, the significant upregulation of *BKI1* in autotetraploid apple (Fig. 5a,b) could lead to dwarf plants.

### The relationship between auxin and the autotetraploid dwarf phenotype

IAA biosynthesis is still not completely understood. Several IAA biosynthesis pathways have been proposed over the past years, but none of these pathways have been completely defined^49^. Although we were unable to find any genes involved in IAA biosynthesis that were differentially expressed, we found that the IAA content was significantly decreased in autotetraploid plants (Table 4). The concentration of IAA is positively associated with stem elongation and height^19^,^50^. A study of pea described ‘*nana*’, a dwarf genotype containing no detectable GAs with a very low IAA content, and ‘Slender’, a tall genotype with no detectable GAs but a high IAA content^19^. The relationship between IAA content and stem height in transgenic plants was also reported. Overexpression of *GmDof17-1* from soybean in tobacco plants caused a dwarf phenotype, and the IAA content was decreased^51^. Exogenous application of IAA could significantly promote plant elongation. Thus, the decreased IAA content in autotetraploid plants could lead to the dwarf phenotype.

*AUX1, TIR1, AUX/IAA* and *ARF* are key genes involved in the auxin signal transduction pathway^39^. Auxin promotes the interaction of *TIR1 and AUX/IAA*^52^. When auxin levels are low, Aux/IAA proteins bind to auxin response factors (ARFs) and repress ARF function^53^. In this study, *AUX1, TIR1* and *MdARF3* were found to be downregulated in the autotetraploid (Fig. 5a,b). This finding suggested that the auxin signal transduction pathway was significantly affected, which could lead to suppression of plant development.

### Possible crosstalk between auxin and BR

Both IAA and BR promote cell expansion. Microarray studies have revealed that as many as 40% of all BR-induced genes are also upregulated by auxin^54^,^55^. Auxin and BR promote *Arabidopsis* hypocotyl (embryonic stem) elongation in a synergistic and interdependent fashion^55^. The synergistic interaction between BR and auxin may be due to the activity of ARFs^56^. BIN2 phosphorylates ARF2, thus preventing ARF2 from binding to the promoters of some auxin-responsive genes in *Arabidopsis*^57^ Our results suggest that the genes involved in determining the dwarf phenotype in autotetraploid apple have roles in the auxin signal transduction pathway and in the BR biosynthesis and signal transduction pathways. Notably, based on our DGE data, BIN2 was found to be significantly upregulated, while *MdARF3* was downregulated (Supplementary information); these results were subsequently verified by qRT-PCR analysis. BIN2 and MdARFs may be key players in the possible crosstalk between auxin and BR.

### A hypothesis to explain the dwarf phenotype of autotetraploid apple

After genome doubling, differential expression of genes involved in auxin and BR biosynthesis pathways leads to decreased levels of both auxin and BR. In the absence of endogenous IAA, Aux/IAA proteins bind to ARFs and repress their function^53^, interrupting the auxin signal transduction pathway. This represses cell elongation and leads to a dwarf phenotype in autotetraploid apple. In the same way, when endogenous BR is absent, BKI1 maintains BRI1 in an inactive state through a direct interaction, and BIN2 phosphorylates BZR1 to inhibit its DNA binding activity^58^. Thus, the BR signal transduction pathway is interrupted and cell elongation is repressed, leading to a dwarf phenotype in autotetraploid apple. At the same time, BIN2 may phosphorylate ARFs, preventing ARFs from binding to the promoters of some auxin-responsive genes^57^. And after genome doubling, the upregulation of miR390 level (Figs 6c and 7a) leads to upregulation of *MdTAS3s* expression (Fig. 7b), which in turn causes downregulation of *MdARF3* expression (Figs 4 and 5). Overall, this leads to partial interruption of the IAA signal transduction pathway. Our study provides important insights into the molecular mechanisms underlying dwarfism in autopolyploid apple plants.

## Materials and Methods

### Plant material

Autotetraploid plants were induced *in vitro* from the diploid apple (*Malus* × *domestica*, 2*n *= 2*x = *34) cultivar ‘Hanfu’ by colchicine treatment of leaves. Sixty regenerated plants were obtained, and of these, 12 individual plants were preliminarily classified as tetraploids based on an investigation of chromosome numbers^59^. Of the 60 regenerated plants, 20 individual plants were obtained as diploid controls. Both of the autotetraploid and diploid apples are the same background. All plants were grown in tissue culture and subsequently transplanted to the field at the same time in 2008. The plants were managed under identical conditions without pruning.

### Plant height measurement

For the analysis of plant height, we selected five typical diploid individuals and five typical tetraploid individuals to serve as one biological replicate. A total of three replicates were performed. The plants were selected and measured at the Shenyang Agricultural University test site after annual growth had ceased in autumn. Measurements were taken annually from 2008 to 2012. The tests were taken at that time, can show the growth height of a year clearly. Plant height was analysed using the GLM model in SAS 9.2, with years as a repeated measure.

### Shoot culture of 5-year-old autotetraploid and diploid apple plants

At Feberuray 20^th^ 2013, a total of 30 nodal segments from each autotetraploid and diploid apple plant at the dormancy stage were immersed in water under nature light at room temperature. After two weeks, lateral buds had sprouted. The buds were washed with running water for 1 h, surface sterilized by immersion in 70% (v/v) ethanol for 30 s, and soaked in a solution of HgCl_2_ (0.1%, w/v) for 10 min. After the terminal shoots were rinsed three times with sterile distilled water, the shoot tips (1 mm) were excised and inoculated on MS medium^60^. The medium was supplemented with 1 mg L^−1 ^BA, 0.2 mg L^−1^ NAA and 0.5 mg L^−1^ GA_3_ to establish *in vitro* shoot cultures. For shoot proliferation culture, stem cuttings (1 cm in length) from the *in vitro* shoots were placed vertically on MS medium supplemented with 0.3 mg L^−1^ BA, 0.2 mg L^−1^ IAA and 0.1 mg L^−1^ GA_3_ and subcultured at 5-week intervals. Cultures were maintained at 25 °C under a 16-h photoperiod with light provided by cool white fluorescent lamps (36 mmol m^−2^ s^−1^).

Forty clones of each of the rooted plantlets were transplanted into pots containing a 2:2:1 mixture of perlite, vermiculite and vermicopost, and they were maintained in a greenhouse. The plants were managed under identical conditions, and plant height was assessed 5 months after transplantation.

### Observation by light microscopy

When the new primary shoot reached about 10 cm, the shoot tip of autotetraploid and diploid counterparts were collected in Mid-Apri of 2013. Paraffin sections were made according to the method of Chen *et al*.^61^. The slides were mounted with synthetic resin, and they were observed and photographed under a light microscope (Nikon ECLIPSE 80i; Tokyo, Japan).

### RNA extraction and quality determination

Total RNA was isolated using the modified CTAB method as described by Chang *et al*.^62^, and the RNA samples were treated with DNase I (TaKaRa, Japan) for 4 h. The integrity of the RNA samples was assessed with an Agilent 2100 Bioanalyzer (Agilent Technologies, Palo Alto, USA).

### DGE library construction and sequencing

Two typical individual clones of each ploidy apple were selected for DGE analysis. The library construction and sequencing were taken by the shoot tips, when the new primary shoot reached about 10 cm of autotetraploid and diploid counterparts in Mid-Apri of 2010. Each sample was sequenced twice, and a total of 4 samples were used for sequencing. The samples for transcript analysis were prepared using Illumina’s kit following manufacturer’s recommendations. Briefly, 6 μg total RNA was used for mRNA capture with magnetic oligo (dT) beads. The first and second cDNA strands were synthesized, and the stranded bead-bound cDNA was subsequently digested with *NLaIII*. The 3′-cDNA fragments attached to the oligo (dT) beads were ligated to the Illumina GEX *NLaIII* Adapter 1, which contained a recognition site for the endonuclease *Mmel* for cutting 17 bp downstream of the recognition site (CATG) to produce tags with adapter 1. After removing 3′ fragments with magnetic beads precipitation. An Illumina GEX adapter 2 was introduced at the site of *Mmel* cleavage. The resulting adapter-ligated cDNA tags were amplified using PCR-primers that were annealed to the adaptor ends for 15 cycles. The 85 base fragments were purified and recovered by 6% polyacrylamide Tris-borate-EDTA gel. The final quality of tagged sequences was checked by Agilent 2100 Bioanalyzer. The two constructed tag libraries underwent Illumina proprietary sequencing chip (flow cell) for cluster generation through situ amplification and were deep sequenced using Illumina Genome Analyzer. Image analysis, base calling, and quality calibration were performed using the Solexa Automated Pipeline, after which the raw data (tag sequences and counts) were produced.

### Data processing and digital tag profiling

Raw sequence reads were filtered by the Illumina pipeline. The 3′ adaptor sequence was removed from raw sequences. All low quality tags such as short tags (<21 nt), empty reads, and singletons (tags that occurred only once) were removed. The remaining high quality sequences were mapped to apple genome (apple genome, http:// www.rosaceae.org/) and NCBI nr database using an E-value cut-off of 10^−5^. The KEGG pathways annotation was performed using Kyoto Encyclopedia of Genes and Genomes database.

### Evaluation of DGE libraries

Statistical analysis of the frequency of each tag in the different cDNA libraries was performed to compare gene expression in the two materials. Statistical comparisons were performed with custom scripts using the method described by Audic *et al*.^63^. The false discovery rate (FDR) was used to determine the P value threshold in multiple tests and analyses. We used FDR < 0.001 as the threshold to determine significance of gene expression differences. In this study, we used FDR < 0.001 and | log2 Ratio | ≤1 as the thresholds to determine significant differences in gene expression. For pathway enrichment analysis, we mapped all differentially expressed genes to terms in the KEGG database and searched for significantly enriched KEGG terms compared to the genome background.

### Microarray analysis

The RNA was taken by the shoot tips, when the new primary shoot reached about 10 cm of autotetraploid and diploid counterparts in Mid-Apri of 2010. The microarrays, which were labeled with Cy3 and Cy5, were constructed by CapitalBio (Beijing, China). On two miRNA chips, 1278 spots were used to provide three replicates of 426 known miRNAs from *Arabidopsis*, rice, maize, sorghum, and other plants along with various controls as described by Li *et al*.^64^. The procedures for microarray hybridization and data evaluation were previously described in detail^65^. Expression analysis of miRNAs was performed using SAM software. A fold change >2 was considered to indicate differential expression.

### Reverse transcription

We performed reverse transcription of nine genes according to the methods of Ma *et al*.^66^. The primers used are shown in Table S1. Reverse transcription of miR390 and *MdTAS3-1* was performed according to the method of Li *et al*.^64^, and the primers are shown in Table S1.

### qRT-PCR analysis

To verify the data obtained from DGE, three typical individual clones from diploid plants and three typical individual clones from autotetraploid plants were selected for qRT-PCR analysis, and three biological replicates were performed for each ploidy. The analysis was performed using young shoot tips obtained in 2010 and 2012. qRT-PCR was performed with an iQ5 Real-Time PCR system (Bio-Rad) in a final reaction volume of 20 μl containing 1 μl of cDNA, 10 μl of 2.5 × RealMaster Mix SYBR Green (TianGen, Beijing, China), and 200 nM forward and reverse primers (Online Resource 2). The reactions were incubated in a 96-well plate (Applied Biosystems) at 95 °C for 3 min, followed by 40 cycles of 95 °C/10 s and 60 °C/30 s.

To detect the difference in expression of miR390 and *MdTAS3-1* between diploids and autotetraploids, qRT-PCR was performed with an iQ5 Real-Time PCR system (Bio-Rad) in a final reaction volume of 20 μl containing 1 μl of RT product, 8 μl of 2.5 × RealMaster Mix (TianGen, Beijing, China), 1 μl of 20 × probe enhancer solution, 0.5 μl of 10 μm TaqMan Probe, and 1 μl of 10 μM forward and reverse primers (Table 1). The reactions were incubated in a 96-well plate (Applied Biosystems) at 95 °C for 10 min, followed by 40 cycles of 95 °C/15 s and 60 °C/60 s.

Relative fold changes in gene and miRNA expression were calculated using the comparative Ct (2^−ΔΔCt^) method with 18S rRNA as the endogenous control. In each biological replicate the genes and template control were carried out in triplicate. The threshold cycle (Ct) was defined as the cycle number at which the fluorescence signal exceeded the fixed threshold.

### Exogenous supplementation of tissue culture plants with IAA and EBR

Five shoot tip cuttings (1 cm in length) from each autotetraploid and diploid apple plant grown in tissue culture were placed vertically on MS medium, which was used in the shoot proliferation culture described above as a control. Five shoot tips from autotetraploid plants and five shoot tips from diploid plants were placed vertically on proliferation medium supplemented with 0.4 mg L^−1^ IAA and 0.1 mg L^−1^ EBR. Cultures were maintained at 25 °C under a 16-h photoperiod with light provided by cool white fluorescent lamps (36 mmol m^−2^ s^−1^). Four weeks later, we compared the heights of plants grown with and without exogenous hormone supplementation.

### Hormone content measurement by HPLC-ESI-MS/MS

When the new primary shoot reached about 10 cm the shoot tip of autotetraploid and diploid counterparts were collected in Mid-Apri of 2010 and 2012. Five typical diploid individuals and five typical autotetraploid individuals were selected for hormone content measurement. We used three biological replicates of each ploidy. Quantification of endogenous IAA and GA_1_ was performed as described previously^67^. Quantification of endogenous BR (BL) was also performed as described previously^68^.

## Additional Information

**How to cite this article**: Ma, Y. *et al*. Involvement of Auxin and Brassinosteroid in Dwarfism of Autotetraploid Apple (*Malus* × *domestica*). *Sci. Rep.* **6**, 26719; doi: 10.1038/srep26719 (2016).

## Supplementary Material

Supplementary Table 1

Supplementary Dataset 1

## Figures and Tables

**Table 1:** Heights of diploid and autotetraploid apple plants (cultivar ‘Hanfu’) from 2008 to 2012.

Ploidy	Plant height (cm)				
	2008	2009	2010	2011	2012
2*x*	61.80 ± 6.78*	123.40 ± 5.99*	187.88 ± 5.58*	199.20 ± 3.99*	208.20 ± 2.92*
4*x*	35.90 ± 3.60	71.40 ± 6.67	121.00 ± 6.08	135.53 ± 2.73	155.20 ± 6.69

Each value is the mean and standard deviation of five replicate plants. *Means in the same row followed by different letters were significantly different at *р* < 0.05. 2*x*: diploid; 4*x*: autotetraploid.

**Table 2:** Categorization and abundance of sequence tags.

Summary		2*x*–1	2*x*–2	4*x*–1	4*x*–2
Raw data	Total	5,973,749	4,706,099	5,955,350	4,940,994
	Distinct tags	351,039	310,224	342,858	310,404
Clean tags	Total number	5,788,249	4,539,994	5,757,002	4,761,962
	Distinct tag number	168,845	147,135	148,314	134,039
All tags mapping to a gene	Total number	450,4847	3,582,944	4,209,307	3,548,525
	Total % of clean tags	77.83%	78.92%	73.12%	74.52%
	Distinct tag number	91,448	8,0564	7,6043	6,9051
	Distinct % of clean tags	54.16%	54.76%	51.27%	51.52%
Tags that unambiguously map to a gene	Total number	1,762,062	1,461,855	1,656,118	1,552,632
	Total % of clean tags	30.44%	32.20%	28.77%	32.60%
	Distinct tag number	51,523	46,232	42,586	40,066
	Distinct % of clean tags	30.51%	31.42%	28.71%	29.89%
All tag-mapped genes	Number	84,771	75,054	77,570	67,968
	Percentage of reference genes	52.67%	53.01%	48.20%	48.00%
Unambiguous tag-mapped genes	Number	25,798	24,932	22,465	21,950
	Percentage	16.03%	17.61%	13.96%	15.50%
Unknown Tag	Total number	509,789	957,050	601,348	1,213,437
	Total % of clean tags	8.81%	21.08%	10.45%	25.48%
	Distinct tag number	37,246	66,571	34,341	64,988
	Distinct % of clean tags	22.06%	45.24%	23.15%	48.48%

Clean tags are those remaining after filtering to remove low-quality tags from the raw data. Distinct tags are different types of tags. Unambiguous tags are the remaining clean tags after removal of tags that map to reference sequences from multiple genes.

**Table 3:** Expression profiles of genes involved in hormone biosynthesis and signal transduction pathways in apple.

Gene ID (Apple Genome Data)	TPM-2*x*	TPM-4*x*	log2 (4*x*/2*x*)	P-Value	FDR	Annotation
*AUX1* MDP0000885425	15.20	4.34	−1.81	1.57E–09	1.97E–08	Auxin signal transduction
*MdARF3* MDP0000179650	46.99	20.84	−1.17	1.22E–14	2.88E–13	Auxin signal transduction
*DWF4* MDP0000144510	50.79	22.58	−1.17	1.22E–15	3.06E–14	BR biosynthesis
*BKI1* MDP0000124873	2.76	11.29	2.03	6.15E–08	6.47E–07	BR signal transduction
*BSK* MDP0000092692	20.90	5.38	−1.96	7.38E–14	1.58E–12	BR signal transduction
*BIN2* MDP0000295137	6.91	21.02	1.61	6.14E–11	8.85E–10	BR signal transduction
*GA2ox* MDP0000137705	1.21	0.01	−6.92	0.007983	0.002949	GA biosynthesis
*DELLA* MDP0000205622	31.79	10.94	−1.54	5.89E–15	1.42E–13	GA signal transduction
*GID2* MDP0000126528	22.46	7.30	−1.62	9.63E–12	1.50E–10	GA signal transduction

**Table 4:** Plant hormone levels in diploid and autotetraploid apple plants (cultivar ‘Hanfu’) assayed in 2010 (3 years old) and 2012 (5 years old).

Ploidy	IAA content (ng/g FW)		GA_1_ content (ng/g FW)		Brassinosteroid content (ng/g FW)	
	2010	2012	2010	2012	2010	2012
2*x*	88.03 ± 20.71*	79.39 ± 10.70*	0.36 ± 0.02	0.11 ± 0.01	0.75 ± 0.04*	0.70 ± 0.03*
4*x*	60.03 ± 11.98	42.08 ± 2.42	0.32 ± 0.01	0.08 ± 0.01	0.15 ± 0.06	0.48 ± 0.08

Each value is the mean and standard deviation of five replicate plots with three trees per plot. *Means in the same row followed by different letters were significantly different at р < 0.05.
