# Supplementary material for: Involvement of Auxin and Brassinosteroid in Dwarfism of Autotetraploid Apple (Malus × domestica)
Source: Sci Rep. 2016 May 24;6:26719. doi: 10.1038/srep26719 (PMC4877651; doi:10.1038/srep26719)
Supplement: Supplementary Table 1 [file srep26719-s1.pdf]

Involvement of Auxin and Brassinosteroid in Dwarfism of Autotetraploid Apple (*Malus* × *domestica*)

Yue Ma[1], Hao Xue[1], Lei Zhang[1], Feng Zhang[1], Chunqing Ou[2], Feng Wang[3], Zhihong Zhang[1*]

[1]College of Horticulture, Shenyang Agricultural University, 120 Dongling Road, Shenyang 110866, China

[2]Research Institution of Pomology, Chinese Academy of Agricultural Sciences, Xingcheng, Liaoning 125100, China

[3]College of Plant Protection, Shenyang Agricultural University, 120 Dongling Road, Shenyang 110866, China

* corresponding author email zhang_sau@163.com, +86-24-88342261

Sumplemental Table 1 Primers used to perform quantitative RT-PCR for assay of gene and miRNAs

Name	Sequence (5'-3')		
AUX1	RT primer:		Oligo d(T)18; random primer (9 mer)
	PCR primer	Forward:	GATGCATGAGACCAAGAGCA
		Reverse:	GGTGGTTTCTCCACAGCATT
MdARF3	RT primer:		Oligo d(T)18; random primer (9 mer)
	PCR primer	Forward:	AGAAAGGGACTGTGGTGGTG
		Reverse:	CATTGCCTCAACATCCTCCT
GA2ox	RT primer:		Oligo d(T)18; random primer (9 mer)
	PCR primer	Forward:	GAGAGCACACAGACCCACAA
		Reverse:	TGGTCCCCCAAAATAAATCA
DELLA	RT primer:		Oligo d(T)18; random primer (9 mer)
	PCR primer	Forward:	ATCCAGCGACTTGGAGCTTA
		Reverse:	GGCTGCCATGTAACCAAACT
GID2	RT primer:		Oligo d(T)18; random primer (9 mer)
	PCR primer	Forward:	ATGATCGGCAAGGAAACAAC
		Reverse:	TGGGTAATATGGAGGGTGGA
DWF4	RT primer:		Oligo d(T)18; random primer (9 mer)
	PCR primer	Forward:	TGATGATCCTTTGGCATTCA
		Reverse:	AGACCGGGAGTTCGGACTAT
BKI1	RT primer:		Oligo d(T)18; random primer (9 mer)
	PCR primer	Forward:	GCAGGCATGAAGAAGAAAGG
		Reverse:	ATCAATGGCGAAAGAAGGTG
BSK	RT primer:		Oligo d(T)18; random primer (9 mer)
	PCR primer	Forward:	CCCCGTTTCAAAAACAGAAA
		Reverse:	CGGCACCTTCCTCATCTTTA
BIN2	RT primer:		Oligo d(T)18; random primer (9 mer)

	PCR primer	Forward:	CGGGTAACCAGTTTTCTGGA
		Reverse:	CTGCACATTTGTTGGTTTGG
miR390	RT primer:		CTCAACTGGTGTCGTGGAGTCCGGCAATT CAGTTGAGGGCGCTAT
	PCR primer	Forward:	ACACTCCAGCTGGGAAGCTCAGGA
		Reverse:	AACTGGTGTCGTGGAG
	Probe		FAM-TTCAGTTGAGGGCGATAT-TAMRA
MdTAS3-1a	RT primer:		CTCAACTGGTGTCGTGGAGTCCGGCAATT CAGTTGAGAAGACCTT
	PCR primer	Forward:	AGGTGGTGGTTGATGGGAAT
		Reverse:	AACAAAGACAACAGACCCGG
	Probe		FAM-TTCAGTTGAGAAGACCTT-TAMRA
18s	RT primer:		Oligo d(T)18; random primer (9 mer)
	PCR primer	Forward:	GTAGTCATATGCTTGTCT
		Reverse:	GAATGATGCGTCGCCAGCACAAAGG
	Probe		FAM-CAGAAGTCGGGATTTGTTGC-TAMRA

FAM 6-carboxy-fluorescein, reporter fluorophore, TAMRA 6-carboxy-tetramethylrhodamine, quencher fluorophore
